# Mean platelet volume is associated with the presence of left atrial stasis in patients with non-valvular atrial fibrillation

**DOI:** 10.1186/1471-2261-13-40

**Published:** 2013-06-10

**Authors:** Rui Providência, Ana Faustino, Luís Paiva, Andreia Fernandes, Sérgio Barra, Joana Pimenta, Joana Trigo, Ana Botelho, António M Leitão-Marques

**Affiliations:** 1 Centro Hospitalar e Universitário de Coimbra, Serviço de Cardiologia, Coimbra, Portugal; 2 Faculty of Medicine, University of Coimbra, Coimbra, Portugal

**Keywords:** Atrial fibrillation, Stroke, Left atrial appendage thrombus, Mean platelet volume

## Abstract

**Background:**

Mean platelet volume has been associated with stroke in patients with atrial fibrillation. However, its role as a predictor of left atrial stasis, assessed by transesophageal echocardiography, in patients with non-valvular atrial fibrillation has not yet been clarified.

**Methods:**

Single center cross-sectional study comprising 427 patients admitted to the emergency department due to symptomatic atrial fibrillation and undergoing transesophageal echocardiogram evaluation for exclusion of left atrial appendage thrombus before cardioversion. All patients had a complete blood count performed in the 12 hours prior to transesophageal echocardiogram. Markers of left atrial stasis were sought: left atrial appendage thrombus, dense spontaneous echocardiographic contrast and low flow velocities in the left atrial appendage. The presence of at least one of the former markers of left atrial stasis was designated left atrial abnormality. Binary logistic multivariate analysis was used for obtaining models for the prediction of transesophageal echocardiogram endpoints.

**Results:**

Left atrial appendage thrombus was found in 12.2%, dense spontaneous echocardiographic contrast in 29.7%, low flow velocities in 15.3% and left atrial abnormality in 34.2%. Mean platelet volume (exp β = 3.41 p = 0.048) alongside with previous stroke or transient ischemic attack (exp β = 5.35 p = 0.005) and troponin I (exp β = 5.07 p = 0.041) were independent predictors of left atrial appendage thrombus. Mean platelet volume was also incorporated in the predictive models of dense spontaneous echocardiographic contrast, low flow velocities and left atrial abnormality, adding predictive value to clinical, echocardiographic and laboratory variables.

**Conclusions:**

These findings suggest that mean platelet volume may be associated with the presence of markers of left atrial stasis, reinforcing a likely cardioembolic mechanism for its association with stroke in patients with non-valvular atrial fibrillation.

## Background

Thromboembolism is one of the most feared complications of atrial fibrillation (AF) [1]. It may arise due to AF in the course of time or it may be facilitated by procedures like cardioversion or percutaneous AF ablation, when a thrombus is present in the left atrium. Therefore, before undergoing risk procedures like catheter ablation or cardioversion of AF a pre-procedural transesophageal echocardiogram may be advisable in order to minimize post-procedure thromboembolic complications [2,3].

The possibility of using biomarkers for thromboembolic risk stratification of patients with atrial fibrillation is a field of growing interest. The role of mean platelet volume (MPV) as a predictive marker of stroke in patients with AF has been recently suggested by Ha and colleagues [4]. In this investigation, MPV was shown to add incremental predictive value to the clinical variables present in the CHADS_2_ score.

Very recently, it was also shown in a case–control study that stroke patients with AF displayed higher MPV levels than patients with AF without stroke history of stroke [5]. These authors established a cut-off level of MPV > 9.4 fL for this association (OR 4.021 p < 0.001).

However, the precise mechanism underlying this relationship (cardiac embolism or peripheral thrombosis due to increased platelet reactivity) is not completely understood. It is thought that at least 90% of thrombi in patients with AF originate in the left atrial appendage [6]. Other markers of left atrial stasis, like dense spontaneous echo contrast (DSEC) and low flow velocities (LFV) in the left atrial appendage [7], are also known to be associated with thromboembolic complications in patients with AF.

The association of MPV with the different markers of left atrial stasis (i.e. its role as a marker of increased risk of cardioembolic stroke) in patients with non-valvular AF has not yet been addressed.

### Aim

To test the accuracy of MPV for predicting markers of left atrial stasis, detected while using transesophageal echocardiogram, in patients with non-valvular AF.

## Methods

### Study population

A single center cross-sectional study was conducted using the following inclusion and exclusion criteria for the definition of the assessed population:

Inclusion criteria:

– All patients undergoing echocardiographic assessment, comprising both transesophageal and transthoracic echocardiogram, due to symptomatic AF leading to admission to the Emergency Department during a 36 months period.

Step-wise exclusion criteria:

– Lack of assessment of MPV in the 12 hours immediately before the echocardiographic assessment.

– Valvular AF, defined as rheumatic heart disease, prosthetic heart valve or previous valve repair and moderate to severe mitral or aortic valve stenosis or regurgitation.

– Presence of ongoing infection.

– Diagnosis of acute myocardial infarction during the index event or in the previous month.

Among a total of 611 subjects, 507 had performed a complete blood count 12 hours prior to transesophageal echocardiogram and were selected for possible inclusion the purpose of our investigation. Among these, 28 subjects with valvular AF, 49 with concomitant infection and 3 with final diagnosis of acute myocardial infarction were excluded from analysis. Our study population included the remaining 427 patients. All subjects provided their informed consent to undergo the necessary investigations and to allow the usage of their data for research purposes, preserving their anonymity.

Baseline overall group characterization with demographic, anthropometric, clinical, laboratory and echocardiographic data, alongside with information on medication was obtained for all patients. Data was retrospectively retrieved from clinical records (outpatient clinic evaluations, emergency department and hospital ward admissions). This study was conducted with the approval of our Institution’s Cardiology Department Supervisor and Ethics Committee.

### Echocardiographic data

Transthoracic and transesophageal echocardiogram were performed using a GE Vivid 7 echocardiograph alongside with M4S (1.5–4.0 MHz) and 6 T phased array multiplane transesophageal (2.9–7.0 MHz) probes. All examinations were performed by two cardiologists with accreditation in transthoracic and transesophageal echocardiography by the European Society of Cardiology. Transesophageal echocardiogram was performed without anesthesia or sedation in more than 97% of patients. Images were later reanalyzed using the GE Health Care EchoPac Dimension software, PC version 108.1.4. Left atrium volume was measured using the single-plane area length method. On transesophageal echocardiogram, the left atrium and left atrial appendage were imaged in different tomographic planes to detect the presence of LAAT and DSEC. Spontaneous echo contrast was classified according to the classification (1 to 4+) proposed by *Fatkin et al*. [8]. Grade 3+ or 4+ was defined as DSEC. Left atrial appendage flow velocities were assessed with a pulsed Doppler sample placed 1 cm from the entry of the left atrial appendage into the body of the left atrium. Emptying and filling velocities were estimated from an average of five well-defined emptying and filling waves. Patients with emptying and filling velocity ≤20 cm/s were classified as having LFV. The presence of at least one of the previous markers of left atrial stasis (LAAT, DSEC or LFV) was designated as left atrial abnormality (LA ABN).

The cardiologists performing the transthoracic and transesophageal echocardiogram were blinded for the lab results and clinical information of the patients other than the fact that they were in AF and there was need for excluding transesophageal echocardiogram changes that could contraindicate cardioversion.

### Laboratory data

After venous blood was drawn, it was immediately transferred into our hospital’s laboratory using an automatic internal tube transference system directly connected from different parts of the hospital into the laboratory. On average laboratory measures were performed within 15 minutes of venous blood sampling.

Automated blood cell counting was performed using the Cell-Dyn Sapphire Hematology Analyzer from Abbot Diagnostics. Reference range values for complete blood count data according to local calibration from our hospital’s laboratory were: hemoglobin – 13.0 to 17.5 g/dL; leukocytes – 4.0 to 10.0 × 10^3^/μL; platelets – 150 to 400 ×10^3^/μL; plaquetocrit – 16.7 to 29.3%; MPV 8.17 to 9.65 fL; platelet distribution width (PDW) 14.7 to 17.4%.

### Prediction of transesophageal endpoints

PASW Statistics version 18.0 was used for descriptive and inferential statistical analysis. Comparisons were performed according to the presence/absence of all markers of left atrial stasis. Chi-square was used for nominal variables and Student’s t-test was used for comparison of continuous variables, where appropriate; the Levene’s test was used in order to check the homogeneity of variance; equivalent non-parametric tests were used when Kolmogorov-Smirnov was in favor of absence of normal distribution. Results with p < 0.05 were regarded as significant.

Univariate analysis was performed using the chi-square test. Predictors from univariate analysis were used for obtaining logistic regression models (using the backward stepwise method likelihood ratio; probability for stepwise = 0.10) that could predict all the transesophageal echocardiogram endpoints: LAAT, DSEC, LFV and LA ABN.

Continuous variables such as left ventricle ejection fraction (LVEF), and MPV were converted into ordinal variables and then used in the logistic regression analysis. Established cutoff points according to data available in the literature were: ≥ 55% vs. < 55% [9] and < 40% vs. ≥ 40% [10] for LVEF; indexed left atrial volume ≥ 60 ml/m^2^ vs. < 60 ml/m^2^[10]; MPV > 9.4 fL vs. ≤ 9.4 fL [5]; troponin I > 0.012 ng/mL vs. non-detectable [11]; body mass index ≥ 27.0K g/m^2^ vs. < 27.0 Kg/m^2^[12]. When cut-off values were not available in the literature, receiver operating characteristic (ROC) curves were traced and using their coordinates we were able to define the optimal cutoff point (Youden index). The Hosmer-Lemeshow summary statistic was used to assess the goodness-of-fit of the models.

## Results

The patients’ baseline clinical, echocardiographic and laboratory characterization is shown on Table 1. Average CHADS_2_ and CHA_2_DS_2_-VASc values were 2.1 ± 1.2 and 3.7 ± 1.7, respectively. Only 147 patients (34.4%) were under oral anticoagulation and 73 of these (49.7%) had an INR ≥ 2.0. In 38.6% (n = 165) of subjects there was no previously known history of AF.

**Table 1:** Population baseline characteristics and sub-analysis according to mean platelet volume

	**Overall**	**MPV ≤ 9.4 fL**	**MPV > 9.4 fL**	**P**
	**(n = 427)**	**(n = 245)**	**(n = 182)**	
Demographics				
Age	68.2 ± 10.7	67.5 ± 11.5	69.0 ± 9.3	0.133
♀	32.1% (137)	31.4% (77)	33.0% (60)	0.736
Body Mass Index (Kg/m^2^)	28.7 ± 5.2	29.2 ± 4.4	28.1 ± 6.0	0.014
Clinical data				
Congestive heart failure	49.4% (211)	45.7% (112)	54.4% (99)	0.076
Hypertension	83.4% (356)	83.3% (204)	83.5% (152)	0.945
Diabetes mellitus	24.6% (105)	24.1% (59)	25.3% (46)	0.777
Stroke or TIA	15.2% (65)	12.2% (30)	19.2% (35)	0.047
Vascular disease^a^	49.6% (212)	46.5% (114)	53.8% (98)	0.135
AF episode duration >1 week	77.8% (332)	74.7% (183)	81.9% (149)	0.078
CHADS_2_ score	2.1 ± 1.2	2.0 ± 1.2	2.3 ± 1.3	0.034
CHA_2_DS_2_-VASc score	3.7 ± 1.7	3.5 ± 1.8	3.9 ± 1.7	0.081
Medication				
Oral anticoagulants	34.4% (147)	33.9% (83)	35.2% (64)	0.782
Enoxaparin	31.1% (133)	32.2% (79)	29.7% (54)	0.570
Antiplatelet agents	48.9% (209)	48.6% (119)	49.5% (90)	0.857
ACE-i or ARB-II	69.6% (297)	65.7% (161)	74.7% (136)	0.045
Statin	41.2% (176)	39.6% (97)	43.4% (79)	0.428
Laboratory assessment				
Haemoglobin (g/dL)	13.8 ± 1.9	13.9 ± 1.9	13.6 ± 1.8	0.089
Leukocytes (10^3^/uL)	7.5 ± 3.1	7.4 ± 2.8	7.8 ± 3.6	0.247
Platelets (10^3^/uL)	222.6 ± 86.2	240.6 ± 95.2	198.4 ± 65.1	<0.001
Plaquetocrit (%)	20.5 ± 7.2	20.8 ± 7.9	20.2 ± 6.2	0.774
MPV (fL)	9.3 ± 1.1			
PDW (%)	16.2 ± 1.3	16.1 ± 1.4	16.3 ± 1.2	0.151
INR	1.4 ± 0.7	1.4 ± 0.7	1.4 ± 0.7	0.583
INR ≥ 2.0	17.1% (73)	16.3% (40)	18.1% (33)	0.624
aPTT time (s)	34.1 ± 7.7	33.0 ± 6.0	35.6 ± 9.5	0.078
Creatinine (umol/L)	111.7 ± 93.9	111.4 ± 107.8	112.1 ± 71.4	0.329
Estimated GFR – MDRD	71.1 ± 27.5	72.4 ± 26.8	69.3 ± 28.5	0.262
CRP (mg/L)	1.7 ± 3.5	1.5 ± 2.7	2.1 ± 4.3	0.213
Troponin I (ng/mL)	0.03 ± 0.07	0.03 ± 0.04	0.04 ± 0.10	0.032
Transthoracic echocardiogram data				
iLAV (ml/m^2^)	60.4 ± 24.5	56.7 ± 21.9	64.7 ± 26.6	0.006
iLVdd (mm/m^2^)	29.6 ± 5.6	28.8 ± 5.5	30.5 ± 5.6	0.010
LVEF < 55%	24.8% (106)	24.5% (60)	25.3% (46)	0.853

Legend: *MPV *Mean platelet volume, *TIA *Transient ischemic attack, *AF* Atrial fibrillation, *ACE-i *Angiotensin converting enzyme inhibitor, *ARB-II* Angiotensin II receptor blocker, *PDW *Platelet distribution width, *INR* International normalized ratio, *CRP*, C reactive protein, *GFR *Glomerular filtration rate, *MDRD *Modified diet in renal disease formula, *LV *Left ventricle, *LAAT *Left atrial appendage thrombi, *DSEC *Dense spontaneous echo contrast, *LFV *flow velocities Lowin the left atrial appendage, *LA ABN *Left atrial abnormality, *aPTT *Activated partial thromboplastin time, *iLAV* Indexed left atrial volume, *iLVdd *Indexed left ventricle diastolic diameter, *LVEF *Left ventricle ejection fraction.

^a ^vascular disease is defined as having at least one of the following: myocardial infarction, peripheral artery disease and complex aortic plaque.

### Mean platelet volume and changes found on transesophageal echocardiogram

Comparisons of patients with MPV ≤ vs > 9.4 fL are shown on Table 1. Patients with MPV > 9.4 fL had a higher prevalence of previous stroke of transient ischemic attack (TIA) (OR = 1.706 _95%_CI 1.004-2.901), a higher CHADS_2_ score and were more frequently medicated with angiotensin converting enzyme inhibitors or angiotensin-II receptor blockers. These patients also displayed lower platelet count levels, higher rise in troponin I levels and a more dilated left atrium and left ventricle.

The following prevalence of markers of left atrial stasis was found on transesophageal echocardiogram: LAAT in 12.2%, DSEC in 29.7%, LFV in 15.3% and LA ABN in 34.2% (Table 2). These were more frequent in patients with MPV > 9.4 fL (Tables 2 and 3).

**Table 2:** Presence of left atrial stasis on transesophageal echocardiogram and sub-analysis according to mean platelet volume

	**Overall**	**MPV ≤ 9.4 fL**	**MPV > 9.4 fL**	**P**
	**(n = 427)**	**(n = 245)**	**(n = 182)**	
**LAAT**	12.2% (52)	9.0% (22)	16.5% (30)	0.019
**DSEC**	29.7% (127)	23.7% (58)	37.9% (69)	0.001
**LFV**	15.3% (54/354)^a^	9.1% (19/208)	24.0% (35/146)	<0.001
**LA ABN**	34.2% (146)	27.3% (67)	43.4% (79)	0.001

Legend: *MPV *Mean platelet volume, *LAAT *Left atrial appendage thrombi, *DSEC *Dense spontaneous echo contrast, *LFV *Low flow velocities in the left atrial appendage, *LA ABN *Left atrial abnormality.

^a ^only 354 subjects had assessment of left atrial appendage flow velocities due to technical or procedural reasons (see Discussion).

**Table 3:** Univariate analysis of parameters eventually associated transesophageal echocardiogram endpoints

**Variable**	**LAAT**		**DSEC**		**LFV**		**LA ABN**	
	**OR**	**P**	**OR**	**P**	**OR**	**P**	**OR**	**P**
	**CI**_**95%**_		**CI**_**95%**_		**CI**_**95%**_		**CI**_**95%**_	
**Congestive heart failure**	1.92	0.031	2.54	<0.001	1.53	0.152	2.77	<0.001
	1.06-3.50		1.65-3.90		0.85-2.74		1.83-4.20	
**Hypertension**	1.32	0.513	1.89	0.043	0.78	0.507	1.81	0.046
	0.57-3.07		1.01-3.54		0.38-1.62		1.00-3.24	
**Diabetes mellitus**	1.58	0.148	1.76	0.016	0.95	0.885	1.83	0.009
	0.85-2.97		1.11-2.80		0.48-1.87		1.16-2.87	
**Age ≥ 65**	1.53	0.218	1.22	0.399	0.97	0.925	1.38	0.153
	0.78-3.02		0.77-1.93		0.51-1.83		0.89-2.16	
**Age ≥ 75**	0.91	0.779	0.58	0.030	0.64	0.194	0.70	0.129
	0.47-1.75		0.36-0.95		0.32-1.27		0.45-1.10	
**Stroke or TIA**	4.12	<0.001	1.72	0.049	1.48	0.314	1.45	0.175
	2.16-7.84		1.00-2.98		0.70-3.17		0.85-2.49	
**Vascular disease**	1.58	0.125	1.80	0.006	1.73	0.066	1.62	0.019
	0.88-2.85		1.18-2.74		0.96-3.12		1.08-2.42	
**Female gender**	1.14	0.677	1.30	0.234	1.68	0.085	1.34	0.178
	0.62-2.10		0.84-2.02		0.93-3.03		0.88-2.04	
**BMI ≥ 27.0 Kg/m**^**2**^	0.85	0.618	1.17	0.497	0.74	0.329	1.05	0.820
	0.45-1.60		0.74-1.86		0.40-1.37		0.68-1.64	
**AF episode duration**	5.31	0.002	2.09	0.009	2.24	0.054	2.29	0.002
**>1 week**	1.62-17.44		1.19-3.67		0.97-5.16		1.34-3.94	
**MPV > 9,4 fL**	2.00	0.019	1.97	0.001	3.14	<0.001	2.04	0.001
	1.11-3.60		1.29-3.00		1.71-5.75		1.36-3.06	
**Platelets ≥ 240 × 10**^**3**^**/uL**	1.83	0.050	1.39	0.142	1.07	0.846	1.39	0.127
	0.99-3.39		0.90-2.15		0.57-2.00		0.91-2.13	
**Troponin > 0.012 ng/mL**	4.92	0.007	3.35	<0.001	3.50	0.008	3.46	<0.001
	1.39-17.41		1.75-6.42		1.40-9.15		1.85-6.48	
**iLVdd ≥ 3.23 mm/m**^**2**^	4.13	<0.001	1.81	0.027	4.66	<0.001	2.40	0.001
	2.01-8.49		1.07-3.06		2.36-9.22		1.43-4.01	
**iLAV ≥ 60 ml/m**^**2**^	4.54	<0.001	4.36	<0.001	6.57	<0.001	4.62	<0.001
	2.10-9.85		2.57-7.40		3.13-13.82		2.76-7.73	
**LVEF < 55%**	2.79	0.001	4.17	<0.001	1.61	0.158	3.76	<0.001
	1.53-5.08		2.62-6.64		0.83-3.12		2.38-5.95	
**LVEF ≤ 40%**	3.00	0.001	5.79	<0.001	1.71	0.167	5.54	<0.001
	1.57-5.74		3.34-10.02		0.79-3.71		3.18-9.68	

Legend: *LAAT *Left atrial appendage thrombi, *DSEC *Dense spontaneous echo contrast, *LFV *Low flow velocities in the left atrial appendage, *LA ABN* Left atrial abnormality, *OR *Odds ratio, *CI *Confidence interval, *TIA *Transient ischemic attack, *BMI *Body mass index, *AF* Atrial fibrillation, *MPV *Mean platelet volume, *iLAV *Indexed left atrial volume, *iLVdd *Indexed left ventricle diastolic diameter, *LVEF* Left ventricle ejection fraction.

After the analysis of ROC curves concerning the discrimination of LAAT, the following cutoff values for platelet count and indexed left ventricle diastolic diameter were found: ≥ 240 × 10^3^/uL (sensitivity = 46.8% and specificity = 67.6%) and  ≥ 3.23 mm/m^2^ (sensitivity = 58.3% and specificity = 75.1%), respectively.

On univariate analysis MPV was a predictor of all markers of left atrial stasis. Congestive heart failure, stroke or TIA, AF episode duration, platelet count, troponin I, iLVdd, iLAV and LVEF were among the other predictors of LAAT on univariate analysis.

Predictors of DSEC, LFV and LA ABN on univariate analysis are shown on Table 3.

### Incremental value of mean platelet volume to other predictors of left atrial stasis

On logistic regression, MPV remained an independent predictor of all markers of left atrial stasis, adding predictive value to clinical variables (stroke or TIA, congestive heart failure and diabetes mellitus), troponin I and echocardiographic parameters (indexed left atrial volume, indexed left ventricle diastolic diameter and LVEF) that were also included in the predictive models (Table 4).

**Table 4:** Binary logistic regression multivariate analysis models for predicting the presence of markers of left atrial stasis

**Endpoint**	**Variable**	**Wald**	**Β**	**Exp β**	**P**	**Hosmer and Lemeshow test**
				**CI**_**95%**_		
**LAAT**	Stroke/TIA	7.812	1.676	5.346	0.005	χ^2^ = 9.093
				1.650-17.317		
	MPV > 9.4 fL	3.899	1.226	3.408	0.048	df = 6
				1.009-11.512		
	Troponin > 0.012 ng/mL	4.157	1.623	5.070	0.041	p = 0.105
				1.065-24.140		
	Constant	0	-0.010	0.990	0.984	
**DSEC**	CHF	7.523	0.845	2.327	0.006	χ^2^ = 1.391
				1.273-4.256		
	MPV > 9.4 fL	4.723	0.642	1.900	0.030	df = 6
				1.065-3.390		
	iLAV ≥ 60 ml/m^2^	16.661	1.221	3.392	<0.001	p = 0.966
				1.887-6.097		
	Constant	6.084	-0.654	0.520	0.014	
**LFV**	MPV > 9.4 fL	4.629	1.257	3.515	0.031	χ^2^ = 2.521
				1.118-11.049		
	iLAV ≥ 60 ml/m^2^	6.275	1.397	4.042	0.012	df = 5
				1.355-12.058		
	iLVdd ≥ 3.23 mm/m2	3.603	1.066	2.905	0.058	p = 0.773
				0.966-8.737		
	Constant	0.444	-0.349	0.705	0.505	
**LA ABN**	MPV > 9.4 fL	4.374	0.977	2.656	0.036	χ^2^ = 5.108
				1.063-6.633		
	iLAV ≥ 60 ml/m^2^	11.504	1.493	4.451	0.001	
				1.878-10.549		
	Troponin > 0.012 ng/mL	8.200	1.387	4.003	<0.001	df = 7
				1.549-10.345		
	DM	4.952	1.065	2.902	0.026	
				1.136-7.418		
	LVEF < 55%	10.316	1.714	5.551	0.001	p = 0.647
				1.950-15.798		
	Constant	17.359	-3.249	0.039	<0.001	

Legend: *LAAT *Left atrial appendage thrombi, *DSEC *Dense spontaneous echo contrast, *LFV *Low flow velocities in the left atrial appendage, *LA ABN* Left atrial abnormality, *CI *Confidence interval, *TIA *Transient ischemic attack, *LVEF *Left ventricle ejection fraction, *MPV *Mean platelet volume, *iLAV *Indexed left atrial volume, *iLVdd *indexed left ventricle diastolic diameter.

MPV was the only variable that could independently predict the presence of all markers of left atrial stasis.

## Discussion

Our data shows that MPV was associated to an increased prevalence of LAAT, DSEC, LVF and LA ABN, echocardiographic markers that are known to associate with cardioembolic stroke and an adverse prognosis in patients with AF. Thus, on logistic regression multivariate analysis MPV was able to add additive predictive value to clinical parameters from the CHADS_2_ score (congestive heart failure, stroke or TIA and diabetes mellitus), echocardiographic parameters (indexed left atrial volume, indexed left ventricle diastolic diameter and LVEF) and other biomarkers (troponin I), as far as prediction of all transesophageal echocardiogram markers of left atrial stasis are concerned.

These results suggest that the recently described increased prevalence of stroke in patients with AF and elevated MPV [5] is possibly due to cardioembolism, which reinforces the importance of our findings in the management of patients with AF:

First, in patients undergoing procedures like cardioversion or AF ablation, a transesophageal echocardiogram (a semi-invasive and highly uncomfortable exam for most patients) is frequently needed in order to rule out the presence of LAAT. By proving the association of MPV with this type of marker, we may contribute to the future development of probabilistic risk scores, combining several variables, that may allow the selection of patients who can be spared this examination (since they are very low risk), or identify those that are very high risk, and therefore must necessarily perform it to prevent thromboembolic complications related to the procedure.

Second, a possible explanation for the association of MPV with stroke could be the increased platelet reactivity leading to peripheral thrombi formation. By showing that this may, in fact, be due to cardioembolism, adequate therapeutic interventions may be pursued or developed for targeting these alterations in patients with AF who test positive for this marker.

Third, novel biomarkers are now being tested in patients with AF in order to improve thromboembolic risk stratification [13]. By demonstrating that MPV possibly associates with stroke due to a cardioembolic mechanism, we provide rationale for its future prospective assessment both separately and as part of risk scores designed for the AF population.

Larger platelets are thought to be younger, more reactive and more thrombogenic [14,15]. MPV seems to reflect both proinflammatory and prothrombotic states [16]. An association of increased MPV with risk factors like diabetes mellitus, hypertension and smoking has been known for some time [17]. An elevated MPV has been found in paroxysmal AF patients when compared to subjects in sinus rhythm [18]. In our study, patients with MPV > 9.4 fL displayed a trend for a higher prevalence of AF episodes lasting for more than one week, which is concordant with these results.

The only investigation assessing the relation between left atrial stasis markers (focusing only on LAAT) and MPV provided negative results towards an association [19]. Still, some points need to be clarified regarding that study: first, it included 18.0% (n = 37) patients with severe mitral stenosis (the number of patients with lower levels of mitral stenosis is not clarified in the manuscript), which may account for the highly unusual elevated prevalence of LAAT (46.8%; n = 96) that was described. Second, the average MPV value was approximately 10.6 fL. That was well over the one we found in our sample (9.3 ± 1.1 fL). Therefore, in our perspective these two studies cannot be appropriately compared.

Based on investigations concerning the composition of spontaneous echocardiographic contrast (thought to be composed of aggregated activated platelets and leucocytes [20]) we are lead into thinking that the association of mean platelet volume with left atrial stasis is not merely due to chance.

Thromboembolic risk stratification of patients with AF is currently based on clinical schemes: the CHADS_2_[21] and CHA_2_DS_2_-VASc score [22]. Still, these classifications have not proved to be as effective in the setting of evaluating patients before AF ablation or cardioversion [23]. In our population, some of the parameters that are part of these classifications, like age, female gender, hypertension and vascular disease, were not independent predictors of left atrial stasis. The same applies to body mass index [12] that displayed no predictive value in our sample.

Troponin I has already been associated both with thromboembolism [13] and with markers of left atrial stasis [11] in AF patients. Concerning indexed left atrial volume, it has been shown to be a predictor of LAAT formation in AF patients [24], but it is still currently lacking a definite proof of its role in thromboembolic risk stratification of patients with AF.

As far as echocardiographic assessment of the left ventricle is concerned, evidence has been more robustly in favor of an association between both LVEF and LAAT [23,25] and between LVEF and systemic embolism [10]. Conversely, evidence is very scarce concerning the role of indexed left ventricle diastolic diameter for the prediction of any thromboembolic endpoint.

To best of our knowledge this is the first report of an association between MPV with markers of left atrial stasis. In face of these results, we may hypothesize that mean platelet volume may be used in the future for assessing patients that are candidates to procedures like catheter ablation or cardioversion of atrial fibrillation. Moreover, these data support the need of its prospective assessment for risk stratification of AF. Due to its wide availability and low cost, mean platelet volume is a potential candidate for this setting.

### Study limitations

A low prevalence of patients undergoing oral anticoagulation was found in our sample. Still, we reinforce that 165 patients (38.6%) had no previous diagnosis of AF. If we consider only patients with previously known AF the prevalence of patients undergoing oral anticoagulation rises steeply to 56.1% (147 out of 262). Therefore, our results can be applied to patients presenting to the emergency department due to symptomatic AF, but probably not to different subsets of patients, like those undergoing percutaneous AF ablation, who have an expected very high prevalence of oral anticoagulation.

Patients over age of 75 displayed a lower prevalence of DSEC on univariate analysis, that was not confirmed after correction for other variables. This may be due to a selection bias, as we only select elderly patients who are very symptomatic and have a preserved biological age for the transesophageal echocardiogram and subsequent cardioversion strategy. As they usually have less risk factors and less severe disease than frail elderly who are directly referred to rate control strategy, this leads to the observed trend for lower prevalence of some markers of left atrial stasis in the transesophageal echocardiogram plus rhythm control strategy.

73 patients (17.1%) had no evaluation of left atrial appendage flow velocities. This was due to technical reasons (echocardiographic data being classified as unsuitable for the accurate assessment of flow velocities) or lack of probe tolerance by the patients (sedation was used in less than 3% of patients). In these subjects, transesophageal echocardiogram was performed without measurement of left atrial appendage flow velocities if the presence of LAAT and DSEC could be excluded right away.

## Conclusions

Our results, using left atrial stasis markers, suggest that mean platelet volume are associated with the presence of left atrial stasis in non-valvular atrial fibrillation. These findings support the likely role of a cardioembolic mechanism in the association between mean platelet volume and stroke in patients with non-valvular atrial fibrillation, reinforcing the possible usefulness of anticoagulation in this patient subset.

Further prospective investigations (derivation and validation studies) will be necessary to confirm the plausibility and role of mean platelet volume as a part of risk models aiming to discriminate the presence of left atrial appendage thrombus in atrial fibrillation patients undergoing procedures like percutaneous ablation or cardioversion.

## Abbreviations

AF: Atrial fibrillation; DSEC: Dense spontaneous echocardiographic contrast; LAAT: Left atrial appendage thrombus; LFV: Low flow velocities; MPV: Mean platelet volume; TIA: Transient ischemic attack; LVEF: Left ventricle ejection fraction.

## Competing interests

The authors declared that they have no competing interest.

## Authors’ contributions

RP designed the study, draft collected data and wrote the first of the manuscript; AF, LP, AF, SB and JP, collected data and critically revised the first draft of the manuscript; JT and AB performed and analyzed all echocardiograms and revised the first draft of the manuscript; AMLM helped in the design of the study, interpretation of data and revised the first draft of the manuscript. All authors read, revised and accepted the final version of the manuscript. All authors read and approved the final manuscript.

## Pre-publication history

The pre-publication history for this paper can be accessed here:

http://www.biomedcentral.com/1471-2261/13/40/prepub
